# Analysis of Environmental Chemical Mixtures and Non-Hodgkin Lymphoma Risk in the NCI-SEER NHL Study

**DOI:** 10.1289/ehp.1408630

**Published:** 2015-03-06

**Authors:** Jenna Czarnota, Chris Gennings, Joanne S. Colt, Anneclaire J. De Roos, James R. Cerhan, Richard K. Severson, Patricia Hartge, Mary H. Ward, David C. Wheeler

**Affiliations:** 1 Department of Biostatistics, School of Medicine, Virginia Commonwealth University, Richmond, Virginia, USA; 2 Occupational and Environmental Epidemiology Branch, Division of Cancer Epidemiology and Genetics (DCEG), National Cancer Institute (NCI), National Institutes of Health (NIH), Department of Health and Human Services (DHHS), Bethesda, Maryland, USA; 3 Department of Environmental and Occupational Health, Drexel University School of Public Health, Philadelphia, Pennsylvania, USA; 4 Division of Epidemiology, Mayo Clinic, Rochester, Minnesota, USA; 5 Department of Family Medicine and Public Health Sciences, Wayne State University, Detroit, Michigan, USA; 6 Epidemiology and Biostatistics Program, DCEG, NCI, NIH, DHHS, Bethesda, Maryland, USA

## Abstract

**Background:**

There are several suspected environmental risk factors for non-Hodgkin lymphoma (NHL). The associations between NHL and environmental chemical exposures have typically been evaluated for individual chemicals (i.e., one-by-one).

**Objectives:**

We determined the association between a mixture of 27 correlated chemicals measured in house dust and NHL risk.

**Methods:**

We conducted a population-based case–control study of NHL in four National Cancer Institute–Surveillance, Epidemiology, and End Results centers—Detroit, Michigan; Iowa; Los Angeles County, California; and Seattle, Washington—from 1998 to 2000. We used weighted quantile sum (WQS) regression to model the association of a mixture of chemicals and risk of NHL. The WQS index was a sum of weighted quartiles for 5 polychlorinated biphenyls (PCBs), 7 polycyclic aromatic hydrocarbons (PAHs), and 15 pesticides. We estimated chemical mixture weights and effects for study sites combined and for each site individually, and also for histologic subtypes of NHL.

**Results:**

The WQS index was statistically significantly associated with NHL overall [odds ratio (OR) = 1.30; 95% CI: 1.08, 1.56; *p* = 0.006; for one quartile increase] and in the study sites of Detroit (OR = 1.71; 95% CI: 1.02, 2.92; *p* = 0.045), Los Angeles (OR = 1.44; 95% CI: 1.00, 2.08; *p* = 0.049), and Iowa (OR = 1.76; 95% CI: 1.23, 2.53; *p* = 0.002). The index was marginally statistically significant in Seattle (OR = 1.39; 95% CI: 0.97, 1.99; *p* = 0.071). The most highly weighted chemicals for predicting risk overall were PCB congener 180 and propoxur. Highly weighted chemicals varied by study site; PCBs were more highly weighted in Detroit, and pesticides were more highly weighted in Iowa.

**Conclusions:**

An index of chemical mixtures was significantly associated with NHL. Our results show the importance of evaluating chemical mixtures when studying cancer risk.

**Citation:**

Czarnota J, Gennings C, Colt JS, De Roos AJ, Cerhan JR, Severson RK, Hartge P, Ward MH, Wheeler DC. 2015. Analysis of environmental chemical mixtures and non-Hodgkin lymphoma risk in the NCI-SEER NHL Study. Environ Health Perspect 123:965–970; http://dx.doi.org/10.1289/ehp.1408630

## Introduction

Risk of non-Hodgkin lymphoma (NHL) is suspected to be associated with several chemicals through occupational or environmental routes of exposure; geographic variation in NHL rates further suggests the importance of environmental risk factors (Hartge et al. 2006). Positive associations have been found with persistent organochlorine chemicals, including polychlorinated biphenyls (PCBs) (Engel et al. 2007a), particularly PCB 180 (Colt et al. 2005; De Roos et al. 2005; Morton et al. 2008), and dichlorodiphenyldichloroethylene (DDE) (Colt et al. 2005; Engel et al. 2007a). An association between NHL overall (Colt et al. 2006) and certain NHL subtypes (Morton et al. 2008) has also been found for residential termite treatment before 1988 (a surrogate for the insecticide chlordane). Several studies have found higher risk of NHL among persons living in areas with industrial emissions to air or industrial waste exposure (Bithell et al. 1994; De Roos et al. 2010; Dreiher et al. 2005; Floret et al. 2003; Franchini et al. 2004; Goldberg et al. 1999; Pronk et al. 2013).

Existing studies of environmental chemical exposures and NHL generally considered only single-chemical risk or total exposure within specific chemical groups, such as PCBs (Colt et al. 2005; De Roos et al. 2005) but did not consider the effects of simultaneous exposure to multiple diverse chemicals or environmental risk factors. Because individuals are exposed to many chemicals simultaneously, it is of particular importance to examine the relationship between chemical mixtures and disease risk. In addition, the analysis of multiple chemical exposures must also consider the inherent correlations among co-occurring environmental chemicals. The complex correlation pattern among chemical exposures and subsequent issue of collinearity has not been directly addressed in studies of NHL or other diseases.

Some studies of environmental factors and disease risk consider many exposures (Everett et al. 2008), sometimes controlling for multiple comparisons in so-called environment-wide association studies (Patel et al. 2010, 2012), but they use separate regression models for each environmental exposure. This type of analysis ignores that environmental exposures may interact (Engel et al. 2007b; Porta et al. 2012). Studies also examine pairwise correlation coefficients between environmental factors (Ioannidis et al. 2009; Patel et al. 2012), but most do not account for the correlation among factors in statistical models. The lack of statistical independence observed among exposures presents challenges to assessing many exposure effects simultaneously in one traditional regression model.

Here, we present an application of the weighted quantile sum (WQS) regression method (Carrico et al. 2014) to estimate an index for 27 correlated environmental chemicals measured in residential carpet dust in a case–control study of NHL. Estimation of chemical weights and the resulting WQS index while considering the correlation between compounds allows us to make generalized inference about the mixture effect and identify the individual chemicals (“bad actors”) most strongly associated with NHL. Because of the design of our study in four geographic regions, the analysis took a site-specific approach in a preliminary effort to consider the effects of spatially varying levels of exposures among chemical mixtures.

## Methods

*Study population*. We conducted a population-based case–control study of NHL in four National Cancer Institute–Surveillance Epidemiology and End Results Program (NCI-SEER) study sites (http://seer.cancer.gov/). The study design has been previously described (Colt et al. 2004; Wheeler et al. 2011). Briefly, the study was conducted in Iowa, Los Angeles County, California, and the metropolitan areas of Detroit, Michigan (Macomb, Oakland, and Wayne counties) and Seattle, Washington (King and Snohomish counties). Eligible cases were 20–74 years of age, diagnosed with a first primary NHL between July 1998 and June 2000, and uninfected with HIV. In Seattle and Iowa, all consecutive cases were chosen. In Detroit and Los Angeles, all African-American cases and a random sample of white (regardless of Hispanic ethnicity) cases were eligible for study, allowing for oversampling of African-American cases. Of the 2,248 potentially eligible cases, 320 (14%) died before they could be interviewed, 127 (6%) were not located, 16 (1%) had moved away, and 57 (3%) had physician refusals. Of the 1,728 remaining cases, 1,321 (76%) participated. Controls (≥ 65 years of age) were selected from Center for Medicare and Medicaid Services files (http://dnav.cms.gov/) or the general population using random digit dialing (< 65 years of age) and were frequency matched to cases by sex, age (within 5-year groups), race, and study site. Of the 2,409 potentially eligible controls, 2,046 were able to be located and contacted, and 1,057 (52%) of these subjects participated. The study was approved by the human subjects review boards at all participating institutions. Written informed consent was obtained from each participant.

Computer-assisted personal interviews were conducted in the home of each participant. Interviewers asked about demographics including race and education, age of the home, housing type, the presence of oriental rugs, pesticide use in the home and garden, residential and occupational histories, and other factors.

*Dust samples and laboratory analysis*. As described in detail previously (Colt et al. 2004, 2005), dust was collected between February 1999 and May 2001 from vacuum cleaners of participants who gave permission (93% of cases, 95% of controls) and who had used their vacuum cleaner within the past year and owned at least half their carpets or rugs for ≥ 5 years [695 cases (57%), 521 controls (52%)]. Dust samples from 682 cases (98%) and 513 controls (98%) were successfully analyzed between September 1999 and September 2001.

Exposure to a mixture of 27 chemicals measured in house dust [5 PCBs, 7 polycyclic aromatic hydrocarbons (PAHs), and 15 pesticides] was of interest. The PCBs were congeners 105, 138, 153, 170, and 180. The PAHs were benz(*a*)anthracene, benzo(*a*)pyrene, benzo(*b*)fluoranthene, benzo(*k*)fluoranthene, chrysene, dibenz(*ah*)anthracene, and indeno(1,2,3-*cd*)pyrene. The pesticides were α-chlordane, γ-chlordane, carbaryl, chlorpyrifos, *cis*-permethrin, *trans*-permethrin, 2,4-dichlorophenoxyacetic acid (2,4-D), DDE, dichlorodiphenyltrichloroethane (DDT), diazinon, dicamba, methoxychlor, *o*-phenylphenol, pentachlorophenol, and propoxur. Extraction and analysis were performed on 2-g aliquots of dust samples using gas chromatography/mass spectrometry (GC/MS) in selected ion monitoring mode. Concentrations were quantified using the internal standard method. Usual detection limits were 20.8 ng/g of dust for α-chlordane, γ-chlordane, DDE, DDT, propoxur, *o*-phenylphenol, PAHs, and PCBs; 42–84 ng/g for chlorpyrifos, diazinon, *cis*-permethrin, dicamba, pentachlorophenol, and 2,4-D; and 121–123 ng/g for carbaryl and *trans*-permethrin. Changes in analytic procedures during the study resulted in increased detection limits for methoxychlor (from 20.7 to 62.5 ng/g). A small proportion of samples weighing < 2 g had detection limits that were higher than the usual detection limits.

The laboratory measurements for the 27 analytes contained various types of ‘‘missing data,’’ primarily when the concentration was below the minimum detection level. To a lesser extent, missing data occurred when there was co-elution between the target chemical and interfering compounds. Chemical concentrations were assumed to follow a log-normal distribution, and data were imputed using a “fill-in” approach to create 10 complete data sets for each of the 27 analytes. Details about the imputation of analyte values have been published previously (Colt et al. 2004; Lubin et al. 2004).

A total of 1,180 subjects with complete dust analysis results and covariate values were included in this analysis. The sample included 508 (43%) controls and 672 (57%) cases, and was predominantly white (88%) with an average age of 60 years (SD = 11.2). Of these 1,180 subjects, 202 (17%) were from the Detroit study site, 340 (29%) from Iowa, 292 (25%) from Los Angeles, and 346 (29%) from Seattle.

*Statistical analysis*. In previous analyses of individual chemicals in the study population overall, we evaluated NHL risk comparing tertiles or other groupings of levels above the detection limit to those with no detectable level of the chemical (Colt et al. 2005, 2006; Hartge et al. 2005). Study site–specific risk estimates were not presented in these publications. Here, we used a weighted quartile sum approach in conjunction with nonlinear logistic regression to evaluate the effect of several chemical exposures together on the risk of NHL. Exposure to a mixture of 27 chemicals measured in house dust was evaluated overall and in study site–specific models. All models were adjusted for sex, age at diagnosis (cases)/selection date (controls), race, and level of education. Age was treated as continuous, race was dichotomized as white or non-white, and education was treated as ordinal (grouped as < 12, 12–15, and ≥ 16 years). In the overall model, we also adjusted for study site.

The WQS method (Carrico et al. 2014) is constrained to have associations in the same direction for chemical exposures and risk, and is designed for variable selection over prediction. WQS regression estimates a weighted linear index in which the weights are empirically determined through the use of bootstrap sampling. The approach considers data with *c* correlated components scored as ordinal variables into quantiles (here, quartiles) that are reasonable to combine (i.e., all chemicals) into an index and potentially have a common adverse outcome. The weights are constrained to sum to 1 and be between 0 and 1, thereby reducing dimensionality and addressing issues associated with collinearity. For this analysis, the *c* = 27 chemical concentrations were scored into quartiles based on the case and control data combined and denoted by *q_i_*, where *q_i_* = 0, 1, 2, or 3 for *i* = 1 to *c*. A total of *B* = 100 bootstrap samples (of the same size as the total sample, *n* = 1,180) were generated from the full data set and used to estimate the unknown weights, w, that maximized the likelihood for *b* = 1 to *B* for the following model


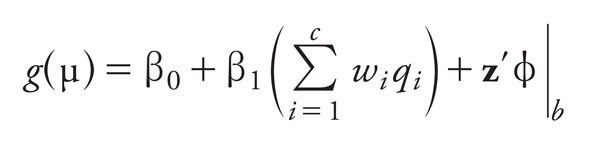
[1]

subject to the constraints *^^c^^*^Σ^*__i__*__=1__*^w^_^i^_*^|^*__b__*  = 1 and 0 ≤ *w_i_* ≤ 1 for *i* = 1 to *c*. In the above equation, *w_i_* represents the weight for the *i*th chemical component *q_i_*, and the term *^^c^^*_Σ_*__i__*__=1__*w_i_q_i_* represents a weighted index for the set of *c* chemicals of interest. Furthermore, **z** denotes a vector of covariates determined prior to estimation of the weights, φ are the coefficients for the covariates in z, and *g*(.) is any monotonic and differentiable link function that relates the mean, μ, to the predictor variables in the right hand side of the equation. Because the outcome variable of interest in this analysis is binary (case status), a logit link was assumed for *g*.

For each bootstrap sample, the *p*-value of β_1_, the parameter estimate for the weighted index, was used to evaluate the statistical significance of the estimated vector of weights (α = 0.10). The weighted quantile score was then estimated as

and *n_B_* is the number of bootstrap samples in which β_1_ was significant. Finally, the significance of the WQS index was determined using the original data set and the model

*g*(μ) = β_0_ + β_1_ WQS + z´φ, [2]

where exp(β_1_) is the odds ratio (OR) associated with a unit (quartile) increase in the weighted sum of exposure quartiles (WQS index).

Weights estimated from the full data set were used to create a WQS index denoted as WQS_F_. In addition to WQS_F_, four site-specific indices [denoted as WQS_D_ (Detroit), WQS_I_ (Iowa), WQS_L_ (Los Angeles), and WQS_S_ (Seattle)] were estimated using data from each site. Differences in the distributions of the chemical concentrations across sites prohibited the use of quantiles based on the full data set in the estimation of site-specific weights; therefore, we used site-specific quartiles based on the combined case–control distribution to estimate site-specific indices. The association between the WQS indices and NHL was examined by testing each index within its respective data set, with statistical significance set at α = 0.05. The primary statistical analysis was performed using one randomly selected imputation data set. A secondary analysis estimated WQS indices for all 10 imputed data sets to assess sensitivity of the results to the data imputation.

We conducted further analyses of major subtypes of NHL: diffuse large B-cell lymphoma (DLBCL), follicular lymphoma, small lymphocytic lymphoma/chronic lymphocytic leukemia (SLL/CLL), marginal zone lymphomas, other lymphomas, and lymphomas where subtype was not specified/unknown [not otherwise specified (NOS)]. Our study primarily included SLL rather than CLL (Morton et al. 2008). Other lymphomas consisted of mantle cell lymphoma, lymphoplasmacytic lymphoma, Burkitt lymphoma/leukemia, mycosis fungoides/Sézary syndrome, and peripheral T-cell lymphoma. We fitted WQS regression models separately for each of these groups to determine whether the mixture effect varied by subtype using all 508 controls in each model.

As a comparison to the WQS regression results, we also conducted single chemical analyses (one-by-one) for all of the data (adjusted for study site) and separately within each study site using study site–specific cut points based on the distributions among cases and controls combined. Models were adjusted for sex, age, race, and level of education. ORs comparing each of the three highest quartiles to the first quartile of exposure were estimated for each individual chemical. Given the exploratory nature of these analyses, no adjustments were made for multiple comparisons.

## Results

Characteristics of the study population are summarized overall and by study site in Table 1. The demographics were similar across the four sites, with the exceptions of race (varying from 73% white in Los Angeles to 99% white in Iowa) and education (individuals with ≥ 16 years of education ranged from 19% in Iowa to 36% in Seattle).

**Table 1:** Characteristics of the study population overall and by study site [*n* (%)].

Characteristic	All sites(*n* = 1,180)	Detroit(*n* = 202)	Iowa(*n* = 340)	Los Angeles(*n* = 292)	Seattle(*n* = 346)
Case status
Control	508 (43)	75 (37)	147 (43)	125 (43)	161 (47)
Case	672 (57)	127 (63)	193 (57)	167 (57)	185 (53)
Age^*a*^ (years)	60 ± 11.2	58 ± 11.3	61 ± 11.4	60 ± 11.2	59 ± 10.8
Sex
Male	631 (54)	114 (56)	181 (53)	163 (56)	173 (50)
Female	549 (47)	88 (44)	159 (47)	129 (44)	173 (50)
Race
White	1,033 (88)	164 (81)	336 (99)	213 (73)	320 (92)
Non-white	147 (12)	38 (19)	4 (1)	79 (27)	26 (8)
Education
< 12 years	106 (9)	24 (12)	33 (10)	30 (10)	19 (5)
12–15 years	741 (63)	123 (61)	244 (72)	171 (59)	203 (59)
≥ 16 years	333 (28)	55 (27)	63 (19)	91 (31)	124 (36)
^***a***^Continuous variable summarized using mean ± SD.					

The distribution of the pairwise Spearman correlations of the chemical concentrations was complex (see Supplemental Material, Figure S1), with pairwise correlations ranging from slightly negative (*r* = –0.15) to nearly perfect correlation (*r* = 0.99 for *cis*- and *trans*-permethrin). Of the 351 unique pairwise correlations, 289 were significant (*p* < 0.05). Correlation among the PAHs ranged from 0.87 to 0.96 (all significant), and correlation among the PCBs ranged from 0.69 to 0.91 (all significant). The correlation among pesticides was generally weaker (84% significant) with an interquartile range of 0.06–0.26. Median concentrations of PCBs were generally similar across the four sites, although the concentration distributions were more positively skewed in Detroit and Seattle than in Iowa and Los Angeles (see Supplemental Material, Figure S2). Chemical concentrations for the PAHs and pesticides varied considerably by site. Concentrations for all seven PAHs were elevated (higher than in other locations) in Detroit, while pesticide concentrations were elevated in Iowa (e.g., carbaryl, 2,4-D, methoxychlor, dicamba) and Los Angeles (e.g., γ-chlordane, *trans*-permethrin, diazinon, propoxur) (data not shown).

The WQS index for the overall study population was significantly associated with NHL (*p* = 0.006, Table 2). More specifically, a quartile increase in the WQS index resulted in an increase of 1.30 [95% confidence interval (CI): 1.08, 1.56] in the odds of NHL in the overall study population. In the site-specific analyses, ORs for a quartile increase in the Detroit (OR = 1.71; 95% CI: 1.02, 2.92), Iowa (OR = 1.76; 95% CI: 1.23, 2.53) and Los Angeles (OR = 1.44; 95% CI: 1.00, 2.08) indices were significantly associated with NHL. The ORs for each of the five WQS indices were generally robust to the analyte imputation (see Supplemental Material, Figure S3). Over the 10 imputations, the average OR for a quartile increase in the WQS index for the study population overall was 1.25, whereas the average site-specific ORs were 1.38 for Detroit, 1.67 for Iowa, 1.61 for Seattle, and 1.45 for Los Angeles. ORs were significant for only 1 imputation in Detroit, and 4 of the 10 imputations in Seattle.

**Table 2:** Associations between NHL and the weighted quantile sum regression index in the study population and in each study site.

Parameter	*n*	OR^*a*^ (95% CI)	*p*-Value
WQS_F_	1,180	1.30 (1.08, 1.56)	0.006
WQS_D_	202	1.71 (1.02, 2.92)	0.045
WQS_I_	340	1.76 (1.23, 2.53)	0.002
WQS_L_	292	1.44 (1.00, 2.08)	0.049
WQS_S_	346	1.39 (0.97, 1.99)	0.071
Abbreviations: WQS, weighted quantile sum index; F, full data set; D, Detroit; I, Iowa; L, Los Angeles; S, Seattle.^***a***^Estimated ORs are associated with a unit increase in the WQS index. All models are adjusted for sex, race, education, and age. The model for the study population (i.e., the full data set) was also adjusted for study site.			

The estimated chemical weights for each WQS index are shown in Table 3. Note that each weight would be 0.037 if all chemicals in the index received equal weight. The most heavily weighted chemicals in the index for the overall data set were PCB 180 [weight (*w*) = 0.32], propoxur (*w* = 0.17), DDE (*w* = 0.08), γ-chlordane (*w* = 0.08), and benzo(*k*)fluoranthene (*w* = 0.07). The weight for PCB 180 was more than eight times the weight expected if all chemicals were equal.

**Table 3:** Weighted quantile sum regression index weights*^a^* estimated in the study population and in each study site.

Chemical	Detroit(*p* = 0.045)^*a*^	Iowa(*p* = 0.002)^*a*^	Los Angeles(*p* < 0.049)^*a*^	Seattle(*p* = 0.071)^*a*^	All sites(*p* = 0.006)^*a*^
PCB 105	0.01	0.07	0.03	0.01	0.02
PCB 138	< 0.005	0.01	0.01	< 0.005	< 0.005
PCB 153	< 0.005	0.01	0.12	0.07	0.02
PCB 170	0.17	0.01	0.01	0.01	0.03
PCB 180	0.18	0.02	0.01	0.14	0.32
Benz(*a*)anthracene	< 0.005	< 0.005	< 0.005	0.09	< 0.005
Benzo(*b*)fluoranthene	< 0.005	< 0.005	0.10	< 0.005	< 0.005
Benzo(*k*)fluoranthene	< 0.005	< 0.005	0.30	0.01	0.07
Benzo(*a*)pyrene	0.07	0.04	< 0.005	0.03	0.06
Chrysene	< 0.005	< 0.005	< 0.005	< 0.005	< 0.005
Dibenz(*ah*)anthracene	< 0.005	0.03	0.03	0.01	0.01
Indeno(1,2,3-*cd*)pyrene	0.03	< 0.005	< 0.005	< 0.005	< 0.005
α-chlordane	0.02	0.07	0.03	0.01	0.04
γ-chlordane	< 0.005	0.12	0.03	0.01	0.08
Carbaryl	0.01	< 0.005	0.03	0.01	0.01
Chlorpyrifos	0.01	0.02	< 0.005	< 0.005	< 0.005
*cis*-Permethrin	0.09	0.01	0.03	0.02	< 0.005
*trans*-Permethrin	0.06	< 0.005	0.01	0.09	0.03
2,4-D	0.05	< 0.005	0.11	< 0.005	< 0.005
DDE	< 0.005	0.11	0.07	0.14	0.08
DDT	0.01	< 0.005	0.01	0.01	< 0.005
Diazinon	0.01	< 0.005	< 0.005	0.02	< 0.005
Dicamba	0.09	< 0.005	0.06	< 0.005	< 0.005
Methoxychlor	0.12	0.01	< 0.005	< 0.005	< 0.005
*o*-Phenylphenol	< 0.005	0.11	< 0.005	0.06	0.04
Pentachlorophenol	< 0.005	0.06	< 0.005	0.09	0.01
Propoxur	0.05	0.30	0.01	0.16	0.17
^***a***^*p*-Value associated with the estimated weighted quantile sum regression index parameter as given in Table 2.					

The chemicals most heavily weighted in the index varied by site (Table 3). PCBs were more heavily weighted in the urban study sites of Detroit, Los Angeles, and Seattle, and pesticides were more heavily weighted in Iowa, an agricultural state. PCB 180 was the most heavily weighted chemical in Detroit (*w* = 0.18), followed by PCB 170 (*w* = 0.17) and the organochlorine pesticide methoxychlor (*w* = 0.12). In Los Angeles, the PAH benzo(*k*)fluoranthene had the highest weight (*w* = 0.30), followed by PCB 153 (*w* = 0.12) and the herbicide 2,4-D (*w* = 0.11). In Seattle, propoxur had the highest weight (*w* = 0.16), followed by PCB 180 (*w* = 0.14) and DDE (*w* = 0.14). The pesticides propoxur (*w* = 0.30), γ-chlordane (*w* = 0.12), DDE (*w* = 0.11), and *o*-phenylphenol (*w* = 0.11) had the highest weights in Iowa. Chemicals that were highly weighted in more than one site included PCB 180 (Detroit and Seattle), propoxur (Iowa and Seattle), and DDE (Iowa and Seattle).

The distributions of the weights for PCB 180, propoxur, and benzo(*k*)fluoranthene, three highly weighted chemicals, differed greatly across the sites (see Supplemental Material, Figure S4). Although PCB 180 was weighted prominently overall, and in Detroit and Seattle, the distribution of its weights was centered near 0 in both Iowa and Los Angeles. Benzo(*k*)fluoranthene had a distribution of weights with a median above 0 only in Los Angeles. The weights for propoxur were mostly distributed above 0 in Iowa, but had a median of 0 for Los Angeles. The distributions of weights for propoxur were similar in Seattle and the full study population.

Of the 672 cases of NHL, 31% were classified as DLBCL, 23% as follicular, 10% as SLL/CLL, 9% as marginal zone, 14% as other, and 13% as NOS. The distribution of cases across subtypes was similar for each site (data not shown). WQS regression results by subtype are shown in Table 4. ORs for a one quartile increase in the index were statistically significant for follicular lymphomas (OR = 1.47; 95% CI: 1.08, 2.00), marginal zone lymphomas (OR = 2.06; 95% CI: 1.25, 3.47), and “other” (OR = 2.26; 95% CI: 1.55, 3.34).

**Table 4:** Associations between NHL subtypes and the weighted quantile sum regression index in the study population.

Parameter	*n*_cases_	OR^*a*^ (95% CI)	*p*-Value
WQS_DLBCL_	207	1.26 (0.94, 1.71)	0.128
WQS_Follicular_	157	1.47 (1.08, 2.00)	0.014
WQS_SLL/CLL_	67	1.26 (0.83, 1.93)	0.273
WQS_MarginalZone_	61	2.06 (1.25, 3.47)	0.006
WQS_Other_	91	2.26 (1.55, 3.34)	< 0.001
WQS_NOS_	89	1.32 (0.91, 1.92)	0.144
Abbreviations: *n*_cases_, number of cases; WQS, weighted quantile sum index; DLBCL, diffuse large B-cell lymphomas; SLL/CLL, small lymphocytic lymphomas/chronic lymphocytic leukemia; NOS, not otherwise specified. ^***a***^Estimated ORs are associated with a unit increase in the WQS index. Models are adjusted for sex, race, education, age, and study site.			

For the individual chemical analyses, the OR for the fourth versus first quartile of exposure for each chemical is listed for the overall analyses in Supplemental Material, Table S1, and for the site-specific analyses in Supplemental Material, Table S2. For the overall study population, PCB 180 was significantly associated with NHL (OR = 1.55; 95% CI: 1.11, 2.17 for the fourth vs. first quartile). Associations between NHL and the remaining PCBs were positive (ORs ≥ 1.20) but not significant (*p*-values ≤ 0.29). In addition, although not significant, the ORs for each of the PAHs were < 1. With respect to the pesticides, the highest quartiles of α-chlordane (OR = 1.40; 95% CI: 0.99, 1.98) and γ-chlordane (OR = 1.35; 95% CI: 0.95, 1.92) were positively associated with NHL, whereas fourth quartile levels of chlorpyrifos (OR = 0.73; 95% CI: 0.52, 1.02), 2,4-D (OR = 0.70; 95% CI: 0.48, 1.03), and dicamba (OR = 0.74; 95% CI: 0.53, 1.04) were inversely associated with NHL.

In the site-specific analyses of individual chemicals (highest vs. lowest quartiles), PCB 180 was significantly associated with NHL in Detroit (OR = 2.87; 95% CI: 1.19, 6.91). In Iowa, associations were positive and significant for α-chlordane (OR = 2.18; 95% CI: 1.15, 4.14), γ-chlordane (OR = 2.25; 95% CI: 1.20, 4.24), DDE (OR = 1.96; 95% CI: 1.05, 3.68), and propoxur (OR = 2.02; 95% CI: 1.09, 3.78); however, associations were negative and significant for 2,4-D (OR = 0.36; 95% CI: 0.19, 0.68) and dicamba (OR = 0.48; 95% CI: 0.26, 0.90). In Los Angeles, benzo(*k*)fluoranthene was significantly associated with NHL (OR = 2.05; 95% CI: 1.04, 4.04). In Seattle, no significant positive associations were found; however, propoxur (OR = 1.53; 95% CI: 0.82, 2.85), DDE (OR = 1.53; 95% CI: 0.83, 2.84), and PCB 180 (OR = 1.53; 95% CI: 0.82, 2.85) were nominally associated with NHL. Finally, 2,4-D (OR = 0.53; 95% CI: 0.29, 0.97) and dicamba (OR = 0.41; 95% CI: 0.22, 0.76) were both significantly inversely associated with NHL in Seattle.

## Discussion

We used weighted quantile sum regression to model the association of a mix of 27 correlated environmental chemicals measured in house dust and risk of NHL in a case–control study in four study centers. We fitted site-specific WQS models and an overall WQS model. We found evidence of an increased risk of NHL associated with an increase in the quantile of the weighted chemical index in the overall study population and in each of the four study sites. These associations were statistically significant for the study population overall and for three of the four study centers (Iowa, Detroit, and Los Angeles), and marginally significant in Seattle.

The most highly weighted chemicals in the overall WQS index were PCB 180, propoxur, γ-chlordane, and DDE. The chemicals most heavily weighted in the site-specific mixture indexes varied by site. Additional chemicals that were relatively highly weighted in site-specific models and associated with an increased risk of NHL included PCB 153 (Los Angeles), PCB 170 (Detroit), 2,4-D (Los Angeles), benzo(*k*)fluoranthene (Los Angeles), methoxychlor (Detroit), and *o*-phenylphenol (Iowa).

By comparison, in single chemical analyses, only PCB 180 was found to be significantly associated with NHL in the study population overall, although γ-chlordane was marginally significant. In prior analyses of these data using a slightly different approach, γ-chlordane and DDE were both associated with significantly increased risks of NHL (Colt et al. 2005, 2006). PCB 180 was the only chemical significantly associated with NHL in analyses of individual chemicals in Detroit. In Iowa, α- and γ-chlordane, DDE, and propoxur were associated with significantly increased risk of NHL. Benzo(*k*)fluoranthene was the only chemical significantly associated with NHL risk in Los Angeles. No chemicals were significantly associated with increased risk in Seattle.

WQS regression highlighted some chemicals that were not significantly associated with NHL in prior analyses or by our single chemical analysis here. These included propoxur in the overall study population, and methoxychlor, *o*-phenylphenol, 2,4-D, PCB 153, and PCB 170 in individual study sites. These chemicals had weights that were several times greater than the value associated with equal weight for all chemicals. In addition, chemicals with non-negligible weights factor positively into the weighted quantile sum index and hence were part of the exposure term found to be significantly associated with NHL in the study population and in three of the four study sites.

Generally, the results from our individual chemical analyses supported the WQS regression findings. The chemicals associated with a significant increase in risk of NHL by individual chemical analyses were also selected as potential risk factors by WQS regression (received non-negligible weights). These include PCB 180 (Detroit), propoxur (Iowa), benzo(*k*)fluoranthene (Los Angeles), and γ-chlordane (Iowa). However, WQS was able to place non-negligible weights on several additional chemicals that were not significantly associated with increased risk in individual chemical analyses. When considered individually, many of these chemicals had elevated ORs that may be potentially meaningful, but were not found to be significantly associated with NHL, likely due to a lack of power. For example, single chemical analysis for propoxur in the full study population resulted in an OR of 1.27 (95% CI: 0.90, 1.79; *p*-value = 0.18) overall. WQS regression selected this chemical as a strong risk factor, giving it a substantial weight (0.17). In Seattle, the single chemical ORs for DDE (OR = 1.53; 95% CI: 0.83, 2.84) and propoxur (OR = 1.53; 95% CI: 0.82, 2.85) were not significant, but these chemicals received WQS weights of 0.14 and 0.16, respectively. In addition, WQS placed zero or negligible index weights on chemicals that were identified as having inverse associations with NHL in the one-by-one analyses (e.g., diazinon and dicamba).

Our findings also show that chemicals identified as important based on a site-specific WQS index may not be identified as important in an index derived from the full data set. Similarly, chemicals identified as important in the index developed from the full data set may not be identified as important in all site-specific indices. These differences are due, in part, to different concentration ranges across sites and overall (see Supplemental Material, Figure S2). The differences may also be due to different sources of these chemicals across study sites or differences in correlations with unmeasured exposures or other factors. For example, PAHs in Los Angeles may be correlated with benzene exposure from traffic, but sources in Detroit may be different. The results illustrate the importance of estimating site-specific weights when developing the chemical mixture index when multiple sites are simultaneously under study.

The main strengths of this analysis are the evaluation of a broad range of environmental chemicals together in one modeling approach, the estimation of the association of a mixture of chemicals with NHL risk, and the estimation of mixture effects by study site. Previous studies have focused on effects of individual chemical exposures. We have used a statistical method that has been shown to have high specificity and adequate sensitivity in identifying important chemicals in regression models in simulation studies (Carrico et al. 2014). In simulation studies based on pairwise correlations of 11 phthalates in the National Health and Nutrition Examination Survey (NHANES; 2005–2008), WQS regression had greater accuracy in identifying the 7 of 11 truly important chemicals (i.e., chemicals set to be related to the outcome) correctly as the correlation of the exposures and the outcome increased from that observed (range of 0.03–0.08) to three times that observed (Carrico et al. 2014). It also showed an improvement in specificity over traditional ordinary regression and popular shrinkage methods (lasso, adaptive lasso, and elastic net). WQS regression tends to place negligible weight on components with no correlation with the outcome.

Single chemical analyses are subject to confounding because of the high degree of correlation among chemical exposure concentrations, but WQS regression allows one to consider exposure to several chemicals simultaneously while accounting for collinearity. Estimation of individual chemical weights enables the identification of potentially harmful chemicals while accommodating the complex correlation observed among exposures. In addition, WQS regression has the advantage of the estimation of a mixture effect and its association with NHL. Because all potentially harmful chemicals (i.e., those receiving non-zero weights) contribute to the estimation of the WQS index, the OR corresponding to the index is interpretable as the increase in risk associated with a quartile increase in the index of the mixture of chemicals, allowing for further inference and insight regarding the potentially harmful effects of exposure to these environmental chemicals.

A limitation of WQS regression is that it cannot identify associations in different directions for the components of the index. However, chemicals in our analysis with inverse associations with NHL in the single chemical analyses were estimated to have negligible weight in the WQS index that had a positive association with NHL. A limitation of the risk analysis is the potential for exposure misclassification from the use of chemical concentrations in house dust as a measure of past exposures. Levels of chemicals in carpet dust do not indicate the source of the chemicals or when they entered the home, nor do they reflect dietary ingestion. However, dust sampling has important advantages over questionnaire- and biologically based approaches. Levels in dust are unaffected by difficulties or biases in recall of past activities, and by factors that may influence body burdens of chemicals such as age, body mass index, and disease status or treatment (Colt et al. 2005). Further, with the exception of the persistent organochlorine chemicals (e.g., PCBs, chlordane, DDT), which are no longer in use, biological measures of many chemicals may reflect only recent exposures due to short half-lives in the body. Carpets act as long-term chemical repositories; hence, chemical concentrations in carpet dust may reflect integrated chemical exposure over the time the carpet was in the home, providing potentially more relevant exposure indicators than a biologic measure of recent exposure. Moreover, studies have found positive associations between chemical concentrations in dust samples and biomarkers of exposure, including serum and house dust levels of PCBs (Knobeloch et al. 2012; Rudel et al. 2008) and lead (Lanphear et al. 1998), and urinary and house dust levels of chromium (Stern et al. 1998).

Another potential limitation of our study was the large number of nonparticipants. However, previous analyses of spatial variation in NHL risk based on eligible nonparticipants and participants in this study population did not substantially change results based on study participants only (Wheeler et al. 2011). In addition, analyses of potential bias in NHL risk associated with census-tract educational level among eligible nonparticipants and participants found a negligible bias between 1% and 8% (Shen et al. 2008). Also, dust samples were collected only from study participants who owned most of their carpets for at least 5 years; therefore, concentrations may not be representative of cases and controls who moved or replaced their carpets within 5 years of the interview.

## Conclusions

We applied weighted quantile sum regression to estimate the association between NHL and an index for 27 correlated environmental chemicals measured in residential carpet dust. The WQS method allowed us to make generalized inference about the chemical mixture effect and identify the individual chemicals most strongly associated with NHL while considering the correlation between compounds. Using WQS regression, we found a positive association between the chemical index and NHL in the overall study population and in each of the four study sites. The WQS analysis also implicated several chemicals as NHL risk factors that were not associated with NHL when evaluated individually. Our results demonstrate the importance of evaluating chemical mixtures when studying cancer risk.

## Supplemental Material

(1.3 MB) PDF
