# Supplementary material for: Analysis of Environmental Chemical Mixtures and Non-Hodgkin Lymphoma Risk in the NCI-SEER NHL Study
Source: Environ Health Perspect. 2015 Mar 6;123(10):965–70. doi: 10.1289/ehp.1408630 (PMC4590749; doi:10.1289/ehp.1408630)
Supplement: (1.3 MB) PDF [file ehp.1408630.s001.acco.pdf]

Environ Health Perspect
doi: 10.1289/ehp.1408630

Note to Readers: ***EHP*** **strives to ensure that all journal content is accessible to all readers. However, some figures and Supplemental Material published in** ***EHP*** **articles may not conform to 508 standards due to the complexity of the information being presented. If you need assistance accessing journal content, please contact ehp508@niehs.nih.gov. Our staff will work with you to assess and meet your accessibility needs within 3 working days.**

Supplemental Material

Analysis of Environmental Chemical Mixtures and Non-Hodgkin Lymphoma Risk in the NCI-SEER NHL Study

Jenna Czarnota, Chris Gennings, Joanne S. Colt, Anneclaire J. De Roos, James R. Cerhan,

Richard K. Severson, Patricia Hartge, Mary H. Ward, and David C. Wheeler

Table of Contents

Table S1. Association between individual chemicals in carpet dust and non-Hodgkin lymphoma for all study sites combined.

Table S2. Association between individual chemicals in carpet dust and non-Hodgkin lymphoma by study site.

Figure S1. Pairwise correlations among the 27 chemical concentrations by type of chemical. There is a high level of correlation within PCBs and PAHs, while the correlations among pesticides and the correlations between chemical groups (intergroup) are lower.

Figure S2. Distribution of chemical concentrations among cases and controls combined in carpet dust by study site for selected chemicals. Boxes extend from the 25th to the 75th percentile, horizontal bars represent the median, and whiskers extend 1.5 times the length of the interquartile range (IQR) above and below the 75th and 25th percentiles, respectively.

Figure S3. Associations[a] between non-Hodgkin lymphoma and weighted quantile sum regression index across the ten imputations for the study population and each study site. [a]Estimated odds ratio and 95% confidence interval (displayed as error bars) associated with a unit increase in the weighted quantile sum regression index. All models were adjusted for

gender, race, education, and age. The model for the study population (i.e., the full data set) was also adjusted for study site.

Figure S4. Distribution of estimated weights for selected chemicals from the weighted quantile sum regression model of non-Hodgkin lymphoma in the study population and each study site. Boxes extend from the 25th to the 75th percentile, horizontal bars represent the median, and whiskers extend 1.5 times the length of the interquartile range (IQR) above and below the 75th and 25th percentiles, respectively. The asterisk denotes the mean weight.

Table S1. Association between individual chemicals in carpet dust and non-Hodgkin lymphoma for all study sites combined.

**Chemical**	**Odds Ratio[a] (95% CI)**	***p*-value**
PCB 105	1.20 (0.87, 1.67)	0.27
PCB 138	1.20 (0.86, 1.68)	0.29
PCB 153	1.31 (0.93, 1.84)	0.12
PCB 170	1.37 (0.98, 1.91)	0.07
PCB 180	1.55 (1.11, 2.17)	0.01
benz(a)anthracene	0.86 (0.57, 1.29)	0.47
benzo(b)fluoranthene	0.83 (0.55, 1.25)	0.37
benzo(k)fluoranthene	0.96 (0.64, 1.43)	0.83
benzo(a)pyrene	0.96 (0.64, 1.44)	0.84
chrysene	0.79 (0.53, 1.17)	0.24
dibenz(ah)anthracene	0.91 (0.61, 1.37)	0.66
indeno(1,2,3-*cd*)pyrene	0.79 (0.52, 1.19)	0.26
α-chlordane	1.40 (0.99, 1.98)	0.06
γ-chlordane	1.35 (0.95, 1.92)	0.09
carbaryl	1.07 (0.77, 1.49)	0.69
chlorpyrifos	0.73 (0.52, 1.02)	0.06
cis-permethrin	0.95 (0.67, 1.34)	0.76
trans-permethrin	0.98 (0.69, 1.39)	0.90
2,4-D	0.70 (0.48, 1.03)	0.07
DDE	1.26 (0.90, 1.76)	0.19
DDT	1.03 (0.73, 1.44)	0.87
diazinon	0.79 (0.56, 1.10)	0.16
dicamba	0.74 (0.53, 1.04)	0.08
methoxychlor	0.90 (0.64, 1.27)	0.55
o-phenylphenol	1.00 (0.71, 1.41)	0.99
pentachlorophenol	1.02 (0.72, 1.45)	0.92
propoxur	1.27 (0.90, 1.79)	0.18

[a]Estimated odds ratios compare the fourth vs. first exposure quartile; quartile cut points were based on the distribution of cases and controls combined. Models were adjusted for gender, race, education, age, and study site.

Table S2. Association between individual chemicals in carpet dust and non-Hodgkin lymphoma by study site.

Chemical	Detroit		Iowa		Los Angeles		Seattle	
	OR[a] (95% CI)	*p*	OR[a] (95% CI)	*p*	OR[a] (95% CI)	*p*	OR[a] (95% CI)	*p*
PCB 105	1.41 (0.61, 3.24)	0.42	1.25 (0.67, 2.32)	0.49	1.20 (0.62, 2.35)	0.59	1.13 (0.62, 2.08)	0.69
PCB 138	1.14 (0.49, 2.68)	0.76	1.24 (0.67, 2.32)	0.49	1.27 (0.65, 2.48)	0.48	1.17 (0.64, 2.16)	0.61
PCB 153	1.27 (0.54, 3.01)	0.59	1.24 (0.66, 2.32)	0.50	1.61 (0.82, 3.16)	0.17	1.25 (0.67, 2.31)	0.48
PCB 170	2.27 (0.95, 5.39)	0.06	1.07 (0.58, 2.00)	0.82	1.27 (0.65, 2.48)	0.49	1.17 (0.63, 2.15)	0.63
PCB 180	2.87 (1.19, 6.91)	0.02	1.23 (0.65, 2.32)	0.52	1.21 (0.62, 2.36)	0.58	1.53 (0.82, 2.85)	0.18
benz(a)anthracene	0.64 (0.26, 1.58)	0.33	0.86 (0.46, 1.59)	0.63	0.88 (0.46, 1.70)	0.71	1.13 (0.61, 2.06)	0.70
benzo(b)fluoranthene	0.73 (0.30, 1.76)	0.48	0.89 (0.48, 1.65)	0.72	1.66 (0.85, 3.25)	0.13	0.93 (0.51, 1.71)	0.81
benzo(k)fluoranthene	0.79 (0.33, 1.91)	0.60	0.94 (0.51, 1.73)	0.83	2.05 (1.04, 4.04)	0.04	1.11 (0.61, 2.03)	0.73
benzo(a)pyrene	0.88 (0.37, 2.13)	0.78	1.05 (0.57, 1.94)	0.88	0.84 (0.43, 1.65)	0.62	1.00 (0.54, 1.83)	0.99
Chrysene	0.71 (0.29, 1.70)	0.44	0.88 (0.48, 1.62)	0.68	1.04 (0.54, 2.00)	0.91	1.22 (0.66, 2.23)	0.53
dibenz(ah)anthracene	0.74 (0.31, 1.79)	0.51	1.17 (0.64, 2.17)	0.61	1.27 (0.65, 2.47)	0.48	1.09 (0.59, 1.98)	0.79
indeno(1,2,3-*cd*)-pyrene	0.90 (0.37, 2.18)	0.82	1.18 (0.63, 2.18)	0.60	0.93 (0.48, 1.82)	0.84	1.01 (0.55, 1.85)	0.97
α-chlordane	1.21 (0.53, 2.76)	0.65	2.18 (1.15, 4.14)	0.02	1.12 (0.58, 2.18)	0.73	0.92 (0.50, 1.68)	0.78
γ-chlordane	0.84 (0.37, 1.94)	0.69	2.25 (1.20, 4.24)	0.01	1.26 (0.65, 2.45)	0.49	0.89 (0.49, 1.63)	0.71
Carbaryl	1.59 (0.68, 3.74)	0.28	0.72 (0.38, 1.33)	0.29	1.16 (0.60, 2.23)	0.66	1.05 (0.57, 1.93)	0.88
Chlorpyrifos	0.82 (0.35, 1.93)	0.65	1.11 (0.60, 2.03)	0.74	0.69 (0.36, 1.34)	0.28	0.69 (0.38, 1.27)	0.23
cis-permethrin	1.60 (0.69, 3.72)	0.28	0.74 (0.40, 1.36)	0.33	1.10 (0.57, 2.11)	0.79	1.06 (0.58, 1.95)	0.85
trans-permethrin	1.19 (0.51, 2.78)	0.69	0.62 (0.33, 1.15)	0.13	0.86 (0.44, 1.65)	0.64	1.07 (0.58, 1.99)	0.82
2,4-D	1.11 (0.47, 2.66)	0.81	0.36 (0.19, 0.68)	< 0.01	1.05 (0.54, 2.04)	0.89	0.53 (0.29, 0.97)	0.04
DDE	0.82 (0.35, 1.94)	0.65	1.96 (1.05, 3.68)	0.04	1.45 (0.75, 2.82)	0.27	1.53 (0.83, 2.84)	0.17
DDT	0.97 (0.41, 2.28)	0.95	1.06 (0.57, 1.97)	0.86	1.13 (0.58, 2.17)	0.72	1.19 (0.64, 2.24)	0.58
Diazinon	0.84 (0.37, 1.92)	0.67	0.82 (0.44, 1.52)	0.53	0.53 (0.27, 1.04)	0.07	0.81 (0.44, 1.48)	0.49
Dicamba	1.07 (0.45, 2.54)	0.88	0.48 (0.26, 0.90)	0.02	0.93 (0.48, 1.81)	0.83	0.41 (0.22, 0.76)	< 0.01
Methoxychlor	1.92 (0.81, 4.57)	0.14	0.98 (0.53, 1.82)	0.95	0.68 (0.35, 1.33)	0.26	0.62 (0.33, 1.15)	0.13
o-phenylphenol	0.52 (0.22, 1.27)	0.15	1.58 (0.83, 3.00)	0.16	0.52 (0.26, 1.02)	0.06	1.03 (0.56, 1.89)	0.93
Pentachlorophenol	0.66 (0.27, 1.62)	0.36	1.24 (0.67, 2.30)	0.50	0.76 (0.39, 1.48)	0.42	1.45 (0.78, 2.73)	0.24
Propoxur	1.06 (0.45, 2.52)	0.89	2.02 (1.09, 3.78)	0.03	0.60 (0.30, 1.17)	0.13	1.53 (0.82, 2.85)	0.18

Abbreviations: OR, odds ratio; *p*, *p*-value

[a]Estimated odds ratios compare the fourth vs. first exposure quartile based on site-specific cut points of cases and controls combined. Models were adjusted for gender, race, education, and age.

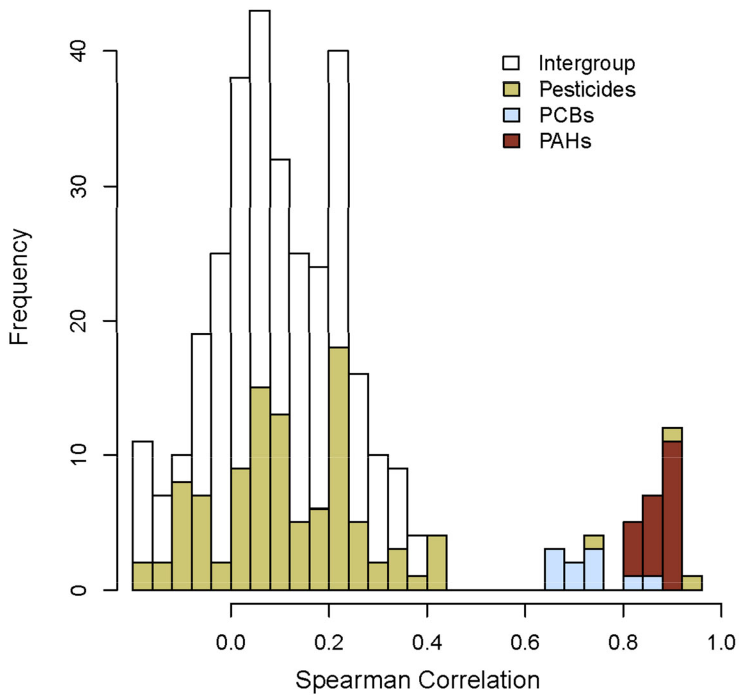

Figure S1. Pairwise correlations among the 27 chemical concentrations by type of chemical. There is a high level of correlation within PCBs and PAHs, while the correlations among pesticides and the correlations between chemical groups (intergroup) are lower.

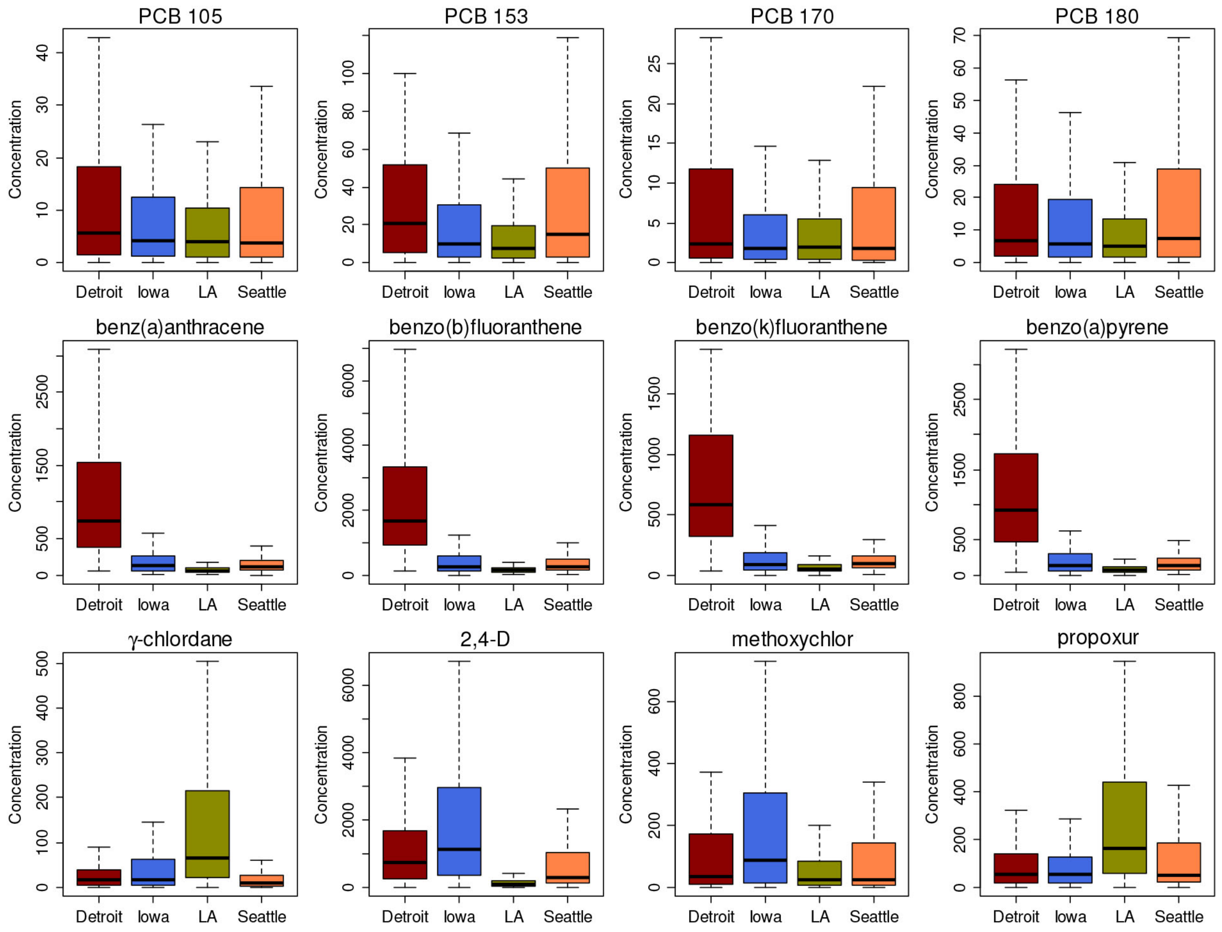

Figure S2. Distribution of chemical concentrations among cases and controls combined in carpet dust by study site for selected chemicals. Boxes extend from the 25th to the 75th percentile, horizontal bars represent the median, and whiskers extend 1.5 times the length of the interquartile range (IQR) above and below the 75th and 25th percentiles, respectively.

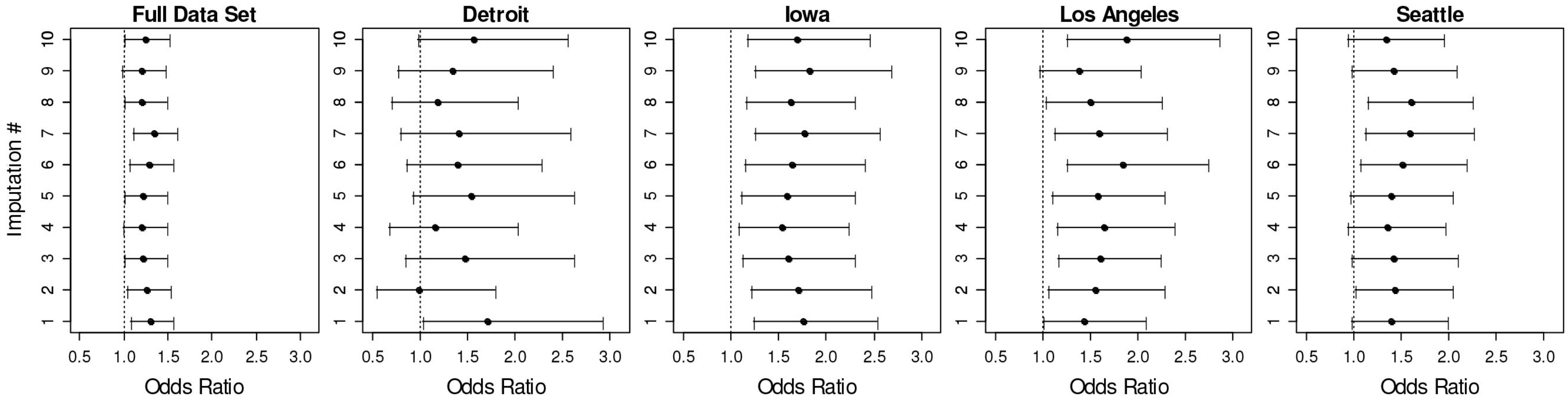

Figure S3. Associations[a] between non-Hodgkin lymphoma and weighted quantile sum regression index across the ten imputations for the study population and each study site. [a]Estimated odds ratio and 95% confidence interval (displayed as error bars) associated with a unit increase in the weighted quantile sum regression index. All models were adjusted for gender, race, education, and age. The model for the study population (i.e., the full data set) was also adjusted for study site.

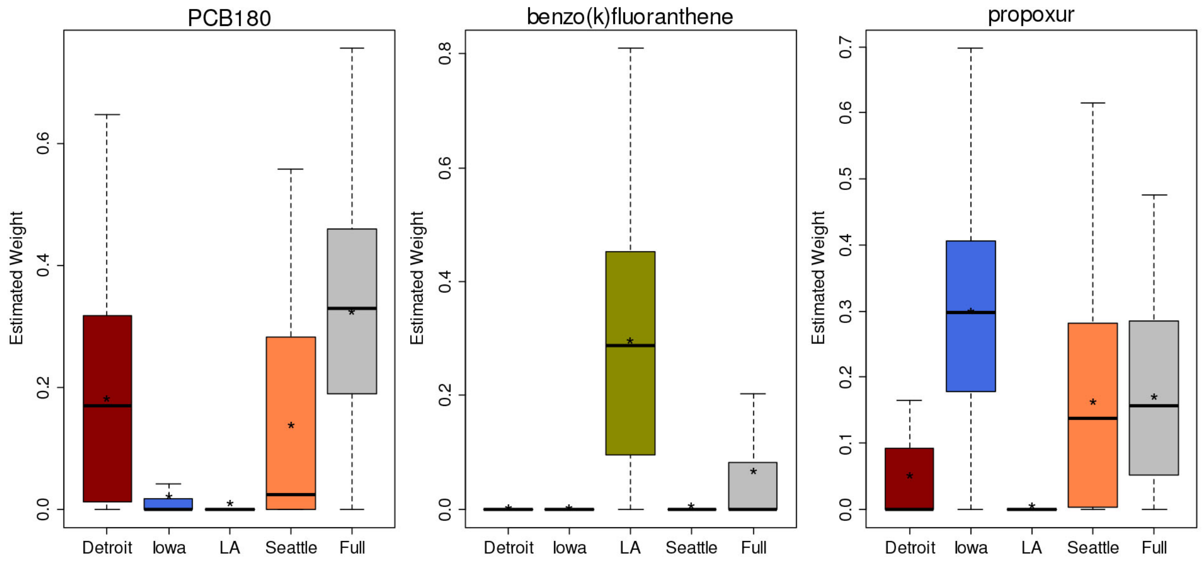

Figure S4. Distribution of estimated weights for selected chemicals from the weighted quantile sum regression model of non-Hodgkin lymphoma in the study population and each study site. Boxes extend from the 25th to the 75th percentile, horizontal bars represent the median, and whiskers extend 1.5 times the length of the interquartile range (IQR) above and below the 75th and 25th percentiles, respectively. The asterisk denotes the mean weight.
